# Feasibility of Skin Water Content Imaging Using CMOS Sensors

**DOI:** 10.3390/s23020919

**Published:** 2023-01-13

**Authors:** Gennadi Saiko

**Affiliations:** Department of Physics, Toronto Metropolitan University, Toronto, ON M5B 1E9, Canada; gsaiko@ryerson.ca

**Keywords:** edema, subepidermal moisture, pressure injury, tissue optics

## Abstract

Pressure injuries (PI) result from pressure-induced damage to the skin and underlying tissues. Currently, Stage I PI are detected using visual skin assessments. However, this visual method is unable to detect skin color changes in persons with darkly pigmented skin, which results in a higher Stage II-IV PI incidence and PI-associated mortality in persons with a darker complexion. Thus, a more objective method of early-stage PI detection is of great importance. Optical spectroscopy is a promising modality for the noncontact diagnosis and monitoring of skin water content, capable of detecting edema and Stage I PI. The scope of the current study is to assess the feasibility of imaging the water content of the skin using Si-based sensors. We have considered two primary cases: the elevated bulk water content (edema) and localized water pool (e.g., blood vessels). These two cases were analyzed using analytical models. We found that detecting the watercontent contrast associated with edema in tissues is within the reach of Si-based sensors. However, although the effect is expected to be detectable even with consumer-grade cameras, with the current state of technologies, their use in real-world conditions faces numerous technical challenges, mainly due to the narrow dynamic range.

## 1. Introduction

Pressure injuries (PI, previously known as pressure ulcers, or PU) result from pressure-induced damage to the skin and underlying tissues, typically above a bony prominence. Most commonly, they can be found among patients in Intensive Care Units (ICU) and residents of Long Term Care Facilities (LTC), where annual incidence rates are reported between 8% and 40% [1]. If managed inadequately, the tissue damage progresses from Stage I (non-blanchable erythema of intact skin) to Stage II (partial-thickness skin loss with exposed dermis) to Stages III and IV. Thus, lower-stage PIs have been suggested as outcome measures of PI prevention care processes in hospitals and LTCs [2,3].

According to NPIAP [4], a Stage I PI is detected using visual skin assessment [5]. However, visual skin assessment is inaccurate at detecting and identifying erythema or Stage I PIs, particularly in individuals with darkly pigmented skin [6,7,8]. For example, one study found [8] that Stage I PIs comprised 32% of all PIs detected in Caucasian residents vs. 0% in African American residents. Therefore, it is plausible that clinicians regularly miss early signs of pressure-induced damage in persons with darker complexion due to the difficulty in observing skin color changes in highly-pigmented skin.

As a result, African Americans are at a higher risk for PI than Caucasians [6,7]. Consequently, they also have higher Stage II-IV PI incidence and PI-associated mortality [9] and are more likely to have multiple PIs [7,8].

Thus, introducing an objective measure that can detect Stage I PI, irrespective of skin tone, is of great importance.

Skin impedance has been recently introduced into clinical practice for the noninvasive diagnosis and monitoring of PI, irrespective of skin tone [10]. However, this methodology has several drawbacks in that it is a single-point measurement, is prone to operator error, and requires contact with the skin. Nevertheless, the underlying principle behind PI detection based on skin impedance measurements can be used to develop other, including noncontact, approaches for PI detection. In particular, it has been established that in the early stages of PI development, inflammation triggered by tissue injury results in increased blood flow to the injured area, causing fluid accumulation in extravascular spaces [11]. Moreover, inflammatory changes and tissue edema occur 3–10 days before visible skin breakdown [12]. Thus, increased tissue water content (often referred to as subepidermal moisture, or SEM) can be a biomarker, capable of detecting erythema and Stage I PI [13] and discriminating between them [14]. Moreover, subepidermal moisture can predict (4.61 days lead time) PI development in patients, including persons with dark skin tones [11]. As a result, the early detection of nonvisible tissue injury using the SEM measurements as an adjunct to the usual PU risk assessment strategies can reduce PU incidence, leading to improved patient outcomes and released productivity [15].

Optical spectroscopy is capable of measuring tissue water content, and can potentially address both drawbacks of the skin impedance-based water content measurements if implemented as a remote modality, e.g., remote imaging.

Moreover, optical spectroscopy techniques can help solve a more general problem associated with water accumulation in tissue, namely edema. As a nonspecific finding, edema is a common clinical sign in various underlying conditions, which can present a significant challenge for the clinician. Thus, the early detection of edema (preferably preclinical, before pitting edema is evident) is of great importance, especially for patients with diabetes, kidney, heart conditions, etc.

Therefore, it would be helpful to develop an optical spectroscopy methodology to examine edema and subepidermal moisture and, ideally, visualize them in various clinical and field settings.

The possibilities for tissue water content imaging were explored previously. In particular, Attas et al. [16] used near-infrared spectroscopic imaging with a wavelength between 960 nm and 1700 nm for *in vivo* skin hydration measurements. However, water absorption is prominent in the near-infrared (NIR) range, which introduces certain limitations for imaging technologies. In particular, strong water bands at 1940 and 1450 nm are out of reach for inexpensive Si-based imaging sensors. 

Nevertheless, the water spectrum in the NIR range has additional features which have the potential to be used in imaging. In particular, there are O-H bond vibrational modes at 760, 970, 1190, 1450, and 1940 nm [17]. Some of these bands can provide information about the local environment of the water molecule. For example, the differentiation between types of water in skin (liquid or bound), based on hydrogen-bonding differences, was observed in the second derivatives of near-infrared reflectance spectra [18]. In particular, water bands associated primarily with lipid bilayers (1880 nm) within the stratum corneum, primary and secondary water of hydration on stratum corneum keratin (1928 and 1910 nm, respectively), and free water below the stratum corneum (1890 nm) were assigned. 

Water absorption bands in NIR have different strengths. In particular, 970 nm is the weakest absorption band, which can be observed *in vivo* (the 760 nm band is much weaker and masked by other tissue chromophores). Absorption at 1190 nm is twice as strong as those at 970 nm (1 and 0.5 cm^−1^, respectively). On the other hand, absorption at the 1450 and 1940 nm bands, 28.6 and 129 cm^−1^, respectively, is much stronger than at 970 and 1190 nm, which makes their penetration depth on a scale of hundreds of micrometers. Thus, it is unlikely that they can sense anything beyond the epidermis layer. Conversely, the weaker absorption at 970 and 1190 nm makes these ranges suitable for water sensing in deeper skin layers, including the dermis and subcutaneous fat. Moreover, they should not be sensitive to the “dryness” of the skin, since the stratum corneum has a low water content and lower thickness compared to the sampling depth.

The 970 nm range is significant as it is within the sensitivity range of the Si-based sensors. Thus, if the hypothesis that the 970 nm range can be used to visualize the water content of tissues is true, then it could significantly reduce the costs of imaging setups.

The scope of the current study is to assess the feasibility of imaging the water content of the skin in the 970 nm range. Our previous work evaluated the feasibility of imaging large blood vessels [19]. However, the moisture imaging at 970 nm can be impacted by the presence of blood components, such as oxyhemoglobin, which has a broad absorption peak centered at 940 nm. This factor was not evaluated in the previous paper [19]; however, it may complicate water imaging. Thus, the impact of blood presence must be analyzed to assess water imaging feasibility comprehensively. Therefore, in the current study, we analyze the effects of the presence of tissue chromophores using analytical models.

## 2. Methods

### 2.1. Tissue Model

In lean individuals, water accounts for 60% of total body weight. However, the water is distributed unequally between various tissues. For example, the water content of the stratum corneum gradually increases from about 10% to about 30% between the surface and deeper layers, followed by an abrupt increase to about 70% deeper in the epidermis [20].

However, such as tissue absorption and scattering are low in the 970 nm range, the effective coefficient is also low (around 0.5 mm^−1^, see follow-up sections), and we can neglect the inhomogeneity of water distribution caused by the epidermis, which is typically thinner than 1 mm even in glabrous skin (0.1 mm in nonglabrous skin). Similarly, all skin microcirculation compartments (capillary, reticular dermis, upper and lower plexuses) have scales smaller than the effective coefficient. Thus, in the 970 nm range, the skin can be considered a homogeneous 2 mm thick layer. Skeletal muscles have a water content similar to the skin (see Table 1). Adipose tissue has a very different water content (see Table 1). However, the adipose content is minimal for most body parts of interest (feet), so we can ignore it. If we only consider the dermis and skeletal muscles, then the tissue, from an NIR optical perspective, can be viewed as a semi-space.

In this semi-space, we can consider two types of anomalies: increased bulk water content (edema) and localized water pool (large blood vessels and other pools of subepidermal moisture), which will result in the inhomogeneity of reflected light. 

To characterize reflected light inhomogeneity, we can introduce the contrast ratio

(1) $$\varepsilon =\frac{I_{b}-I}{I_{b}}$$

where *I* and *I_b_* are the light intensity measured at some point (e.g., over the anomaly or defect) and background (far from the defect), respectively. Assuming the constant illumination of the tissue, Equation (1) can be deduced as a similar equation for tissue reflectance.

#### 2.1.1. Bulk Water Content

This case refers to edema, where a large area may have elevated water content. In particular, we can consider homogeneous water distribution with two different concentrations: *c*_0_ in the normal skin (background) and *c*_1_ in suspected edema (anomaly). Here, *c*_1_ > *c*_0_. To find the contrast ratio for a bulk water content inhomogeneity, we need to calculate the tissue reflectance and convolve it with the light source spectrum.

##### Tissue Reflectance Model

The total reflectance of the tissue consists of two parts: the specular reflection from the surface and the diffuse reflection. The diffuse reflectance of the homogeneous semi-space with mismatched boundaries can be found using diffuse approximation (see, for example, [21]). Thus, we can write

(2) $$r=r_{01}+\left(1-r_{01}\right)\frac{1-2\mu_{eff}\delta }{1+h\mu_{eff}}$$

Here, $\mu_{eff}=\sqrt{\mu_{a}/\delta }$, $\delta =1/3\mu_{tr}$, $\mu_{tr}=\mu_{a}+\mu_{s}(1-g)$, $h=2\delta (1+r_{10})/(1-r_{10})$, *r*_10_ is the coefficient of reflection of diffuse light on the border of tissue and air, where *μ_a_*, *μ_s_*, and *g* are coefficients of absorption, scattering, and anisotropy, respectively, and *r*_01_ is the coefficient of reflection of diffuse light at the border between air and tissue.

#### 2.1.2. Localized Water Pool

To assess the tissue reflectance in the presence of an inhomogeneous distribution of water content (e.g., large blood vessels), we will follow the formalism initially developed in [22]. Here, a perturbation approach was developed for light propagation in turbid tissues with mismatched boundaries. Defects (e.g., blood vessels) were considered negative light sources immersed in homogeneous media, described using diffuse approximation. The model allows for obtaining analytical solutions for point defects [22] and linear defects arranged horizontally or vertically [23]. However, the model can be extended to inhomogeneous water distribution as well.

For the horizontal blood vessel (e.g., vein), the contrast ratio was explicitly found in [23]. The contrast ratio of the linear defect with cross-section *S* and absorption coefficient *δμ_a_*, buried at a depth *Z* measured as the distance y horizontally away from it can be written as

(3) $$\varepsilon (y,Z)=\frac{3\mu_{tr}\delta \mu_{a}S\exp(-\mu_{eff}Z)}{2\pi }(K_{0}(\mu_{eff}{\left(Z^{2}+y^{2}\right)}^{1/2})-K_{0}(\mu_{eff}{\left({\left(2h+Z\right)}^{2}+y^{2}\right)}^{1/2}))$$

where *K*_0_*(t)* is the modified Bessel function of the second kind. Thus, immediately over the blood vessel (*y* = 0), the contrast ratio will be

(4) $$\varepsilon (Z)=\frac{3\mu_{tr}\delta \mu_{a}S\exp(-\mu_{eff}Z)}{2\pi }(K_{0}(\mu_{eff}Z)-K_{0}(\mu_{eff}(2h+Z)))$$

### 2.2. Simulations

To assess the impact of an elevated water content in tissue, we performed simulations of the contrast ratio in both elevated bulk water content and localized water pool scenarios. 

To calculate the reflectance spectra of the tissue, Equation (2) was used. To obtain the bulk tissue’s integrated contrast ratio, the reflectance spectra were convolved with the Gaussian spectrum of a hypothetical LED with a peak wavelength at 980 nm and HWHM = 20 nm. To obtain the integrated contrast ratio for the localized water pool, the spectral dependence of the contrast ratio for the horizontal inhomogeneity (Equation (4)) was convoluted with the Gaussian spectrum of a hypothetical LED with a peak wavelength at 980 nm and HWHM = 20 nm. The simulations were performed with the range of parameters described in the next section (model parameters). Calculations were performed using MathCad 2001 Professional (PTC, Boston, MA, USA). Visualization was performed using MATLAB 9.5 (MathWorks, Natick, MA, USA).

### 2.3. Model Parameters

#### 2.3.1. Absorption

The absorption of the dermis can be modeled as a combination of background (flash)-, oxyhemoglobin-, deoxyhemoglobin-, and water-related absorption.

(5) $$\mu_{a}=\mu_{a,fl}+c_{Hb}(SO2\times \mu_{a,HbO2}+(1-SO2)\mu_{a,RHb})+c\mu_{a,water}$$

Here, *c_Hb_* is the total blood concentration in the dermis, *SO*2 is the blood oxygen saturation, *HbO*2 and *RHb* refer to oxyhemoglobin and deoxyhemoglobin, respectively, and *c* is the tissue water content.

The background absorption of human flesh can be modeled as [24]: $\mu_{a,fl}=7.84\times 10^{7}\lambda^{-3.255}$ [mm^−1^]. Here, the wavelength *λ* is measured in [nm].

Absorption coefficients for oxyhemoglobin and deoxyhemoglobin are well known [25]. Blood typically occupies around 0.2–0.6% of the physical volume of the dermis [26]. However, other groups report much higher values, up to 7% [23]. Thus, we will model blood content in the 1–10% range. Oxygenation of the blood varies from 97–99% for arterial blood to 60% for venous blood. The light samples both arterial and venous compartments in the general case, so the measured *SO*2 is somewhere between these values. As the venous compartment is larger than the arterial, we will approximate the average *SO*2 as 0.7.

Water absorption can be found in numerous sources, as described in [27].

Note that, in the general case, the expression in Equation (5) should also include fat absorption, as fat has an absorption peak at 930 nm. However, this term was omitted based on our assumption that we ignore adipose tissue.

To model the change in the water content in the localized water pool, we can assume that the water content in the pool (e.g., blood vessels) is equal to or close to 1. Thus, for the large blood vessel (vein), we can approximate using

(6) $$\delta \mu_{a}=(0.6\mu_{a,HbO2}+0.4\mu_{a,RHb})+\mu_{a,water}-\mu_{a}$$

where we assume that the blood oxygenation in the vein is around 60%.

#### 2.3.2. Scattering

The reduced scattering coefficient for the dermis and epidermis can be modeled by a power law [28]: $\mu {’}_{s}\propto \lambda^{-k}$, where *k* = 1.3. Thus, we can model the reduced scattering coefficient by selecting a scattering coefficient value at a particular wavelength (e.g., *μ*′_s_ = 5 mm^−1^ at 633 nm [29] for the reticular dermis) as a reference point $\mu {’}_{s}=2.2\times 10^{4}\lambda^{-1.3}$.

#### 2.3.3. Index of Refraction

The rich data available for the index of refraction in biological tissues can be found in [30]. Each skin layer is characterized by its index of refraction, depending, particularly, on the water content. Thus, in the skin, the index of refraction decreases slightly with depth [29] as the water content increases, resulting in almost negligible scattering at the dermis/epidermis interface (<<1%). However, we will ignore this effect and, based on [31], we will set *n* = 1.43 across all layers.

**Table 1:** Initial and expected water content for a 50% increase in IFV for different tissue types.

**Tissue**	***c_i_*, % [32]**	**ECF, % [32]**	***c_f_*, %**
Skin	72	95	79.1
Skeletal muscle	76	16	77.4
Adipose tissue	14	80 (85)	18.5 (18.8)
Connective tissue	80	100	85.7

#### 2.3.4. Water Content

While water is present virtually all body parts, it can be attributed to the intracellular or extracellular spaces. Extracellular space can be divided further into the intravascular plasma volume and the extravascular interstitial volume [32]. Thus, the water content can be characterized by two parameters: an extracellular fluid fraction (*ECF*) of the total water and interstitial fluid volume (*IFV*) [33].

Edema is associated with an increase in *IFV*. Using this observation, we can model water content in various tissues. In particular, if we assume that α is a relative increase in *IFV*, then the final water content (*c_f_*) can be found using initial water content (*c_i_*) and an extracellular fluid fraction (*ECF*) using the following formula:

(7) $$c_{f}=\frac{\left(1+\alpha \right)\times c_{i}\times ECF+c_{i}\times \left(1-ECF\right)}{\left(1+\alpha \right)\times c_{i}\times ECF+\left(1-c_{i}\times ECF\right)}$$

Table 1 summarizes the initial and expected water content for different tissue types [19].

## 3. Results

### 3.1. Tissue Reflectance

**Figure 1:**
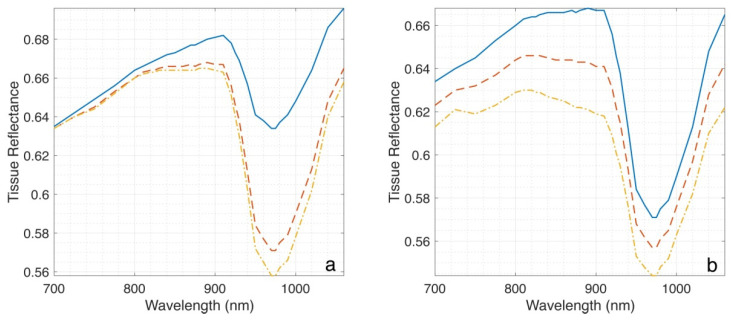
Panel (**a**): tissue reflectance at different water contents: 0.7, 0.8, and 0.3 (red, yellow, and blue lines, respectively). Panel (**b**): tissue reflectance at different blood contents: 0, 0.01, and 0.02 (blue, red, and yellow lines, respectively). *SO*2 was set to 0.7.

We have calculated the reflectance spectrum of the homogeneous tissue for various water contents (See Figure 1a) using Equation (2). In addition, to understand the interplay with oxyhemoglobin absorption in the 940 nm range, we also included the modeling of various total hemoglobin, *c_Hb_* contents (See Figure 1b).

### 3.2. Contrast Ratio of Bulk Water Anomalies

**Figure 2:**
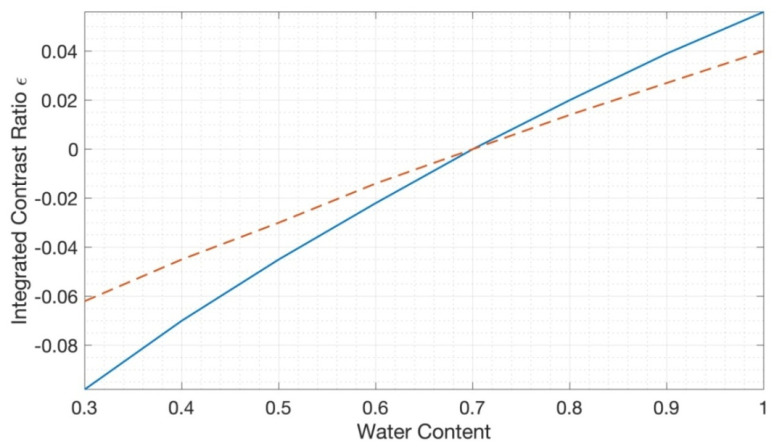
The integrated contrast ratio as a function of water content for several blood content concentrations: *c_Hb_* = 0.01 and 0.1 (solid blue and dashed red lines, respectively). *SO*2 was set to 70%. The normal water content (*c*_0_ = 0.7) was selected as a background.

To emulate the detection of edema, we calculated the integrated contrast ratio of the tissue as a function of water content for several blood content concentrations (See Figure 2). In this case, the reflectance of the homogeneous tissue (Equation (2)) was convolved with the Gaussian spectrum of a hypothetical LED with a peak wavelength at 980 nm and HWHM = 20 nm to obtain the integrated contrast ratio. Here, the area with the normal water content (*c*_0_ = 0.7) was selected as a background.

### 3.3. Contrast Ratio of Localized Pools of Moisture

**Figure 3:**
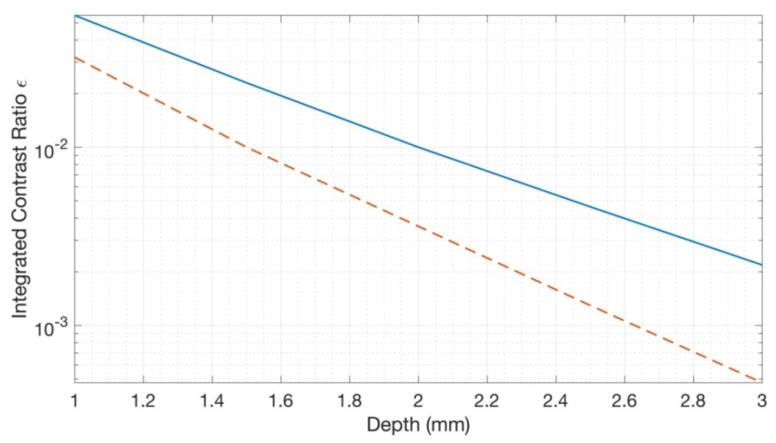
Imaging of large blood vessels. Integrated contrast ratio as a function of the vessel depth (1–3 mm) for several blood content concentrations: *c_Hb_* = 0.01 and 0.1 (solid blue and dashed red lines, respectively). *SO*2 was set to 70%. The normal water content (*c*_0_ = 0.7) was selected as a background. The cross-section of the vessel was set to *S* = 0.1 mm^2^.

We calculated the contrast ratio of large blood vessels as a function of vessel depth for several blood content concentrations (See Figure 3) using Equation (4). The spectral dependence of the contrast ratio for the horizontal inhomogeneity (Equation (4)) was convolved with the Gaussian spectrum of a hypothetical LED with a peak wavelength at 980 nm and HWHM = 20 nm to obtain the integrated contrast ratio.

Note that the contrast ratio in Equation (4) is linear with respect to the cross-section of vessel *S*. Thus, for a larger vessel with a diameter of several mm, the contrast ratio can be 10× larger.

## 4. Discussion

We have presented numerical estimations for skin water content imaging in the 970 nm range. Our estimates show that large inhomogeneities in water content in the skin can be visualized using Si-based sensors. For example, we can expect that detecting even preclinical peripheral edema is possible. In particular, a change in bulk water content from 70% to 80% results in a contrast ratio on the scale of 0.01–0.02 (see Figure 2), which can be detectable even with consumer-grade CMOS-based cameras. 

In addition, from Figure 1 and Figure 2, it can be seen that areas of thick dry skin (e.g., callus) have a noticeably higher reflectance in the 970 nm range that results in significant contrast, which can also be detected by Si-based water content imaging. Thus, the 970 nm range can be used for callus detection, which is of distinct clinical value.

The current results are in line with previous assessments. Calculations based on a model presented in [34] show that we can expect approximately a −0.4% change in reflectance for every 1% increase in the absorption coefficient at 970 nm. In [19], it was estimated that a 5–6% increase in total water content in skin, adipose tissue, and connective tissue (See Table 1) would translate into a −2–2.4% change in tissue reflectance. Our modeling confirms these estimations.

However, water imaging in the 970 nm range faces numerous technical challenges. Firstly, as oxyhemoglobin has a broad peak at 940 nm, blood presence significantly impacts reflectance in the 970 nm range. For example, from Figure 1, it can be seen that changes in blood distribution are comparable with edema-induced changes in reflectance. Moreover, for the case of imaging a large subsurface vessel (veins) (see, for example, [19]), the contrast will most likely be mainly attributed to the presence of oxyhemoglobin.

Secondly, the ambient light presence has a very dramatic effect by narrowing the dynamic range. As we target the changes in reflectance on a scale of several percent, the narrow dynamic range can significantly deteriorate the contrast. This can be illustrated by using the following example. According to Figure 2, the water content change from 70% to 80% will be visible with 1–2% contrast (for 10% and 1% blood content, respectively). Thus, using a consumer-grade camera with an 8-bit depth (minimally resolved single-pixel contrast 1/(2^8^−1) = 0.4%), we can potentially detect water content changes on the scale of 2–4% (for 1% and 10% blood content, accordingly), with realistic values on the scale of 2.9–5.7% (70% of the dynamic range). However, this is true only in the complete absence of ambient light. If ambient light hits the detector, then the sensitivity drops accordingly. For example, for a 2:1 signal mixture (the signal produced by the ambient light is twice as strong as the signal from the LED), the water content limit of detection increases to 8.6–17%. Thus, the elevated water content can be missed entirely (false negative). Consequently, the contribution of ambient light should be substantially suppressed or eliminated.

Theoretical estimations and initial experiments show that the difference in tissue water content can be detected even by consumer-grade 8-bit cameras. However, there are further complications associated with consumer-grade cameras.

The sensitivity of the CMOS sensor at 970 nm is relatively small. For most devices, it is in the 5–10% range of the maximum QE. Moreover, the sensitivity is reduced even further when using a Bayer filter and eliminated entirely when using an IR filter, which is installed on most smartphone cameras. Thus, water imaging with smartphone cameras is quite problematic.

In future work, we plan to perform water imaging in phantoms and patients with edema in clinical settings and compare these results with other methodologies (namely, skin impedance). We also plan to further examine the effect of the presence of blood.

## 5. Conclusions

Our numerical estimations show the feasibility of skin water content imaging in the 970 nm range using inexpensive CMOS sensors. However, even though the effect is expected to be detectable even by consumer-grade cameras, with the current state of technologies, their use in realistic conditions faces numerous technical challenges due to their narrow dynamic range.

## Data Availability

Data is available from the corresponding author upon request.
